# GATA6 identifies an immune-enriched phenotype linked to favorable outcomes in patients with pancreatic cancer undergoing upfront surgery

**DOI:** 10.1016/j.xcrm.2024.101557

**Published:** 2024-05-10

**Authors:** Casper W.F. van Eijck, Francisco X. Real, Núria Malats, Disha Vadgama, Thierry P.P. van den Bosch, Michail Doukas, Casper H.J. van Eijck, Dana A.M. Mustafa, the Dutch Pancreatic Cancer Group (DPCG)

**Affiliations:** 1 Department of Surgery, Erasmus University Medical Centre, Rotterdam, the Netherlands; 2 Genetic and Molecular Epidemiology Group, Spanish National Cancer Research Centre, Madrid, Spain; 3 Epithelial Carcinogenesis Group, Spanish National Cancer Research Centre, Madrid, Spain; 4 Centro de Investigación Biomédica en Red-Cáncer, Madrid, Spain; 5 Department of Medicine and Life Sciences, Universitat Pompeu Fabra, Barcelona, Spain; 6 Department of Pathology and Clinical Bioinformatics, Erasmus University Medical Centre, Rotterdam, the Netherlands; 7 The Tumor Immuno-Pathology Laboratory, Erasmus University Medical Centre, Rotterdam, the Netherlands

**Keywords:** GATA6, immune profiling, pancreatic ductal adenocarcinoma, PDAC, randomized controlled trial, tumor microenvironment

## Abstract

This study underscores GATA6’s role in distinguishing classical and basal-like pancreatic ductal adenocarcinoma (PDAC) phenotypes. Retrospective studies associate GATA6 immunohistochemistry (IHC) expression with survival outcomes, warranting prospective validation. In a prospective treatment-naive cohort of patients with resected PDAC, GATA6 IHC proves a prognostic discriminator, associating high GATA6 expression with extended survival and the classical PDAC phenotype. However, GATA6’s prognostic significance is numerically lower after gemcitabine-based neoadjuvant chemoradiotherapy compared to its significance in patients treated with upfront surgery. Furthermore, GATA6 is implicated in immunomodulation, although a comprehensive investigation of its immunological role is lacking. Treatment-naive PDAC tumors with varying GATA6 expression yield distinct immunological landscapes. Tumors highly expressing GATA6 show reduced infiltration of immunosuppressive regulatory T cells and M2 macrophages but increased infiltration of immune-stimulating, antigen-presenting, and activated T cells. Our findings caution against solely relying on GATA6 for molecular subtyping in clinical trials and open avenues for exploring immune-based combination therapies.

## Highlights

• GATA6^high^ IHC in treatment-naive PDAC favors survival and marks the classical phenotype
• After gemcitabine-based nCRT, the prognostic relevance of GATA6 in PDAC diminishes
• Treatment-naive tumors with varying GATA6 IHC yield distinct immunological landscapes
• GATA6^high^ treatment-naive tumors manifest a potent anti-tumor immune microenvironment

## Graphical abstract

**Figure:**
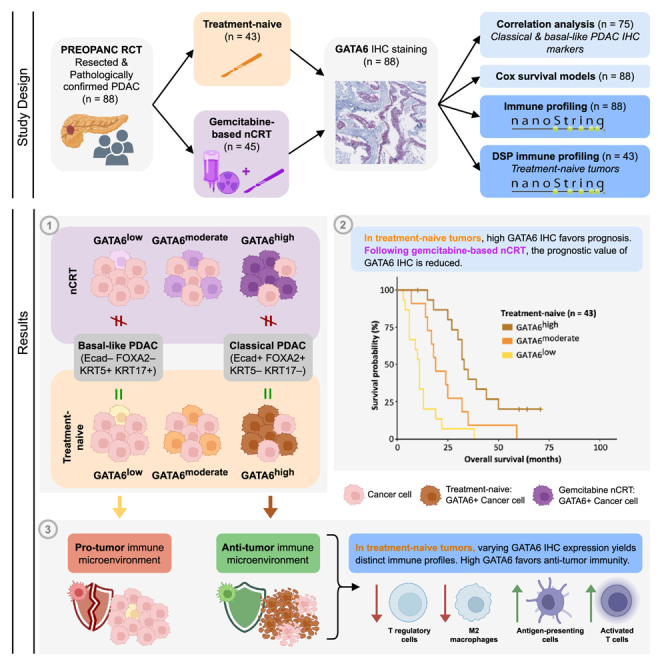

## Teaser

Van Eijck et al. emphasize the prognostic value of GATA6 IHC in treatment-naive PDAC, urging caution with GATA6-based subtyping due to treatment-induced alterations in cellular phenotypes. The association between high GATA6 IHC and an anti-tumor immune environment offers a promising avenue for developing personalized immune therapies to enhance patient outcomes.

## Introduction

Pancreatic ductal adenocarcinoma (PDAC) is an aggressive malignancy with an often dismal prognosis. Most patients present at an advanced stage, limiting the treatment options primarily to systemic multi-drug chemotherapies, which have yielded modest improvements in median overall survival (OS) beyond 1 year.^1^^,^^2^^,^^3^ Patients with resectable PDAC have witnessed notable improvements in 5-year OS rates due to continuous progress in surgical techniques and the use of adjuvant systemic chemotherapy with gemcitabine plus capecitabine or modified 5-fluorouracil (5-FU), folinic acid, irinotecan, and oxaliplatin (mFOLFIRINOX).^4^^,^^5^^,^^6^^,^^7^

Neoadjuvant treatment has garnered attention in managing localized PDAC as it offers potential benefits,^8^ particularly associated with favorable survival outcomes in borderline resectable PDAC. The ESPAC-5 phase 2 trial endorses neoadjuvant chemotherapy over upfront resection or chemoradiotherapy in borderline resectable PDAC.^9^ The PREOPANC randomized controlled trial (RCT) underscored improved OS outcomes with gemcitabine-based neoadjuvant chemoradiotherapy (nCRT), predominantly benefiting borderline resectable, rather than resectable, patients.^10^ Recent findings from the NorPACT-1 RCT challenge the benefit of neoadjuvant FOLFIRINOX compared to upfront surgery in resectable pancreatic (head) cancer.^11^ The lack of consensus on the optimal neoadjuvant approach for PDAC persists, as evidenced by the PREOPANC-2 RCT, which recently indicated no recent improvement in OS for neoadjuvant FOLFIRINOX compared to gemcitabine-based nCRT in patients both with borderline resectable and resectable PDAC.^12^

GATA6 and GATA4 are key regulators of the classical phenotype, with GATA6 assuming a more prominent role.^13^^,^^14^ Low GATA6 RNA expression is associated with the basal-like phenotype and poor survival in patients with PDAC.^13^^,^^15^^,^^16^ Analysis of GATA6 expression alone does not capture the complexity of tumor heterogeneity. However, the dependable and robust assessment of GATA6 expression using immunohistochemistry (IHC) provides a practical advantage over RNA-based subtyping methods. IHC offers an appealing alternative due to its cost effectiveness, widespread availability, and potential for seamless integration into routine clinical practice. Retrospective studies have already demonstrated strong associations between GATA6^high^ histoscores and prolonged survival in treatment-naive patients with resected PDAC^14^^,^^17^ and those with advanced disease.^18^ However, these studies underscore the necessity of prospective investigations to validate their findings. Tumors with GATA6^high^ histoscores respond better to adjuvant 5-FU/leucovorin^13^ than to neoadjuvant mFOLFIRINOX.^19^ In contrast, GATA6 IHC expression is not associated with response to (neo)adjuvant gemcitabine. Although GATA6-defined classical and basal-like subtypes are associated with survival in treatment-naive tumors, these associations diminish following neoadjuvant therapy. This change may result from the co-existence of classical and basal-like phenotypes in post-treatment tumors, making it challenging to classify tumors exclusively as classical or basal. Furthermore, tumor cells can acquire an intermediary phenotype containing both basal and classical features.^19^^,^^20^^,^^21^^,^^22^^,^^23^^,^^24^^,^^25^

GATA6 activates the expression of epithelial genes typically associated with the classical phenotype while restraining epithelial-to-mesenchymal transitions (EMTs).^13^ In addition, GATA6 likely plays a role in immune surveillance, a paramount determinant for countering tumor cell dissemination and mitigating metastatic propensity. The loss of GATA6 in PDAC was associated with defects in antigen processing and presentation pathways and reduced infiltration of activated CD8^+^ T cells.^26^ In addition, a sequencing study revealed an association between basal-like tumors and T cell depletion and the presence of immunosuppressive myeloid cells.^25^ Conversely, a preclinical model has demonstrated that GATA6 knockout augmented T cell-mediated tumor cell killing,^27^ and a negative correlation was observed between GATA6 and the T cell activator PD-L1.^26^ Moreover, another study found that basal-like tumors have a “reactive tumor microenvironment” (TME) with immune-stimulatory and anti-tumoral properties.^28^ Lastly, the intermediary PDAC subtype was linked to increased intra-tumoral and peripheral myeloid cell abundance, mediated by CXCL8.^20^ These conflicting findings underscore the challenge of interpreting the immune pancreatic TME across PDAC subtypes. Deciphering the complex immune ecosystems intrinsic to PDAC tumors with varying GATA6 phenotypes is crucial to address this challenge, given GATA6’s accurate capacity as a distinctive biomarker between the classical and basal subtypes.

This prospective study investigated the prognostic relevance of GATA6 IHC in treatment-naive and gemcitabine-based nCRT-treated patients with PDAC enrolled in the PREOPANC RCT. Furthermore, we conducted transcriptomic and spatial protein profiling to unravel the immune landscapes associated with GATA6 in these tumors, shedding light on their potential contribution to survival outcomes. Our findings offer promising prospects for developing and implementing more effective combination (immuno)therapies.

## Results

### Patient characteristics

**Figure 1:**
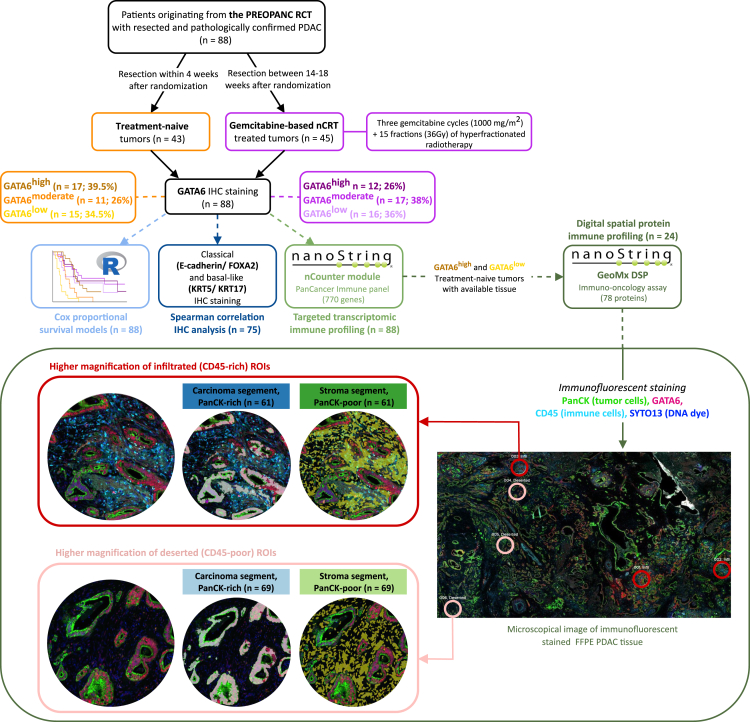
Schematic overview of the methodological steps Schematic overview of the study methodology. Each square represents a methodological step: (1) patient inclusion and clinical procedure, (2) GATA6 IHC staining and tertile grouping, (3) Cox proportional hazards regression modeling, (4) Spearman correlation analysis between GATA6 IHC and classical/basal-like markers, (5) transcriptomic immune profiling using NanoString, and (6) digital spatial protein immune profiling using GeoMx. The digital spatial profiler (DSP) square includes microscopical images of treatment-naive sections illustrating immunofluorescent staining for ROI selection.

Between April 2013 and July 2017, 164 phase 3 PREOPANC RCT participants underwent surgical resection. After excluding 12 patients without pathologically confirmed PDAC, four who did not complete the entire course of gemcitabine-based nCRT, and 23 with insufficient tumor material, the surgical specimens of 125 patients underwent RNA isolation. Following the quality control of tissue RNA and raw gene expression profiles, transcriptomic immune profiling analyses were performed on the specimens of 46 upfront surgery patients and 50 gemcitabine-based nCRT-treated patients.^29^ Of these 96 PDAC tumors, 88 had sufficient tumor material for GATA6 IHC and 75 for co-expression with classical/basal-like phenotype markers. Figure 1 provides a detailed schematic overview of the patient inclusion process and the subsequent methodological steps.

Preoperatively, treatment-naive patients exhibited worse World Health Organization scores (Table 1). Postoperatively, the nCRT-treated group displayed less advanced tumor stages, fewer nodal metastases, less perineural invasion, and a higher rate of R0 resection (Table S1). Following a median follow-up of 73 months, 40 (93%) treatment-naive patients and 30 (67%) nCRT-treated patients had died. OS and progression-free survival (PFS) were prolonged in the nCRT-treated cohort (*p* = 0.002 and *p* = 0.005, respectively). The distribution of GATA6 tertile histogroups differed between the treatment groups, with a greater proportion of GATA6^high^ tumors in the treatment-naive group than the nCRT group (Table 1). The preoperative characteristics were similar among patients in the three GATA6 histogroups across both treatment groups (Table S2). However, in the treatment-naive group, patients with GATA6^high^ tumors exhibited significantly prolonged OS and PFS compared to those with GATA6^low^ tumors (*p* < 0.001).

**Table 1:** Preoperative clinical characteristics of the 88 included patients with resected PDAC

Treatment group	Treatment-naive (*n* = 43)	Gemcitabine-based nCRT (*n* = 45)	*p* value
**Gender, *n* (%)**			

Female	15 (35)	19 (42)	0.5
Male	28 (65)	26 (58)	0.5

Age at diagnosis, years			
Median [min, max]	70 [40, 80]	70 [40, 80]	0.6

BMI, kg/m^2^			
Median [min, max]	20 [20, 30]	30 [20, 40]	1.0

**Diabetes mellitus, *n* (%)**			

No	28 (65)	34 (76)	0.5
Yes	15 (35)	11 (24)	0.5

**Hypertension, *n* (%)**			

No	35 (81)	32 (71)	0.3
Yes	8 (19)	13 (29)	0.3

**History of cancer, *n* (%)**			

No	40 (93)	40 (89)	0.7
Yes	3 (7)	5 (11)	0.7

**History of pancreatitis, *n* (%)**			

No	43 (100)	42 (93)	0.2
Yes	0 (0)	3 (7)	0.2

**Resectability, *n* (%)**			

Borderline resectable	14 (33)	17 (38)	0.7
Resectable	29 (67)	28 (62)	0.7

CA19-9 preoperative, U/mL			
Median [min, max]	300 [1, 6,000]	100 [2, 4,000]	0.2
Missing, *n* (%)	7 (16)	6 (13)	0.2

**Involvement of the SMA, *n* (%)**			

Absent	42 (98)	41 (91)	0.4
Present	1 (2)	4 (9)	0.4

Tumor diameter before nCRT, mm			
Median [min, max]	30 [20, 50]	30 [20, 60]	0.4
Missing, *n* (%)	1 (2)	1 (2)	0.4

Tumor diameter after nCRT, mm			
Median [min, max]	30 [20, 50]	30 [20, 60]	0.6
Missing, *n* (%)	1 (2)	1 (2)	0.6

**Suspicious lymph nodes, *n* (%)**			

Absent	31 (72)	33 (73)	1.0
Present	12 (28)	12 (27)	1.0

**Tumor location, *n* (%)**			

Body/tail	2 (5)	7 (16)	0.2
Head	41 (95)	38 (84)	0.2

**WHO performance status, *n* (%)**			

WHO 0	11 (26)	27 (60)	0.001^a^
WHO 1	32 (74)	18 (40)	0.001^a^

**Response to nCRT (RECIST 1.1), *n* (%)**			

Partial response	0 (0)	5 (11)	–
Stable disease	0 (0)	32 (71)	–
Progressive disease	0 (0)	4 (9)	–
Missing	43 (100)	4 (9)	–

**Hospital, *n* (%)**			

Academical hospital	26 (60)	31 (69)	0.5
General hospital	17 (40)	14 (31)	0.5

WHO, World Health Organization; SMA, superior mesenteric artery; RECIST, response evaluation criteria in solid tumors

a *p* values indicate statistical significance (*p* < 0.05).

### High GATA6 IHC expression is associated with the classical cell phenotype in treatment-naive tumors but not in tumors pretreated with gemcitabine-based chemoradiotherapy

**Figure 2:**
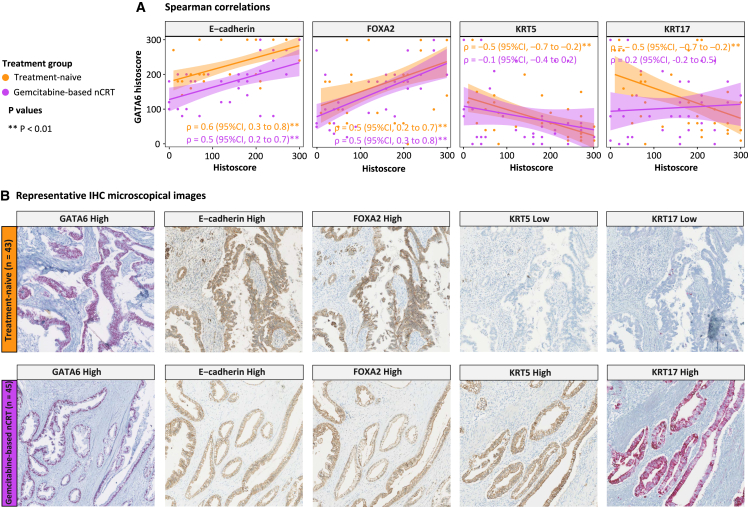
Spearman correlations and IHC analysis of GATA6 and classical (E-cadherin/FOXA2) and basal-like cell (KRT5/KRT17) phenotype markers in resected PDAC tumors (A) Scatterplots illustrating Spearman correlations between the GATA6 histoscores (y axis) and other markers (x axis) stratified by treatment. In treatment-naive tumors, GATA6 shows a significant positive correlation with the classical markers (E-cadherin/FOXA2) and a negative correlation with basal-like markers (KRT5/KRT17). In nCRT-treated tumors, GATA6 shows a significant positive correlation with the classical markers while showing no correlation with basal-like markers. Each dot represents a patient. Correlation coefficients are denoted with ρ with 95% confidence intervals, and ∗∗*p* < 0.01 indicates statistical significance. (B) Microscopical images of GATA6, E-cadherin, FOXA2, KRT5, and KRT17 IHC stains, stratified by treatment, visually demonstrate Spearman correlations. Fast Red chromogen is used for GATA6 and KRT17, while DAB chromogen is used for E-cadherin, FOXA2, and KRT5.

The relationship between GATA6 and classical/basal-like phenotypes was explored through IHC co-expression analysis, assessing GATA6 alongside the classical (E-cadherin and FOXA2) and basal-like (KRT5 and KRT17) phenotypes (Figure 2). Due to sample availability, this analysis was conducted on 75 of 88 PDAC tumors. The clinical characteristics of this patient subset and detailed IHC results are provided in Tables S3 and S4. The significant positive correlation between GATA6 histoscores and the classical markers E-cadherin (treatment naive: ρ = 0.6; 95% confidence interval [CI], 0.3 to 0.8; nCRT: ρ = 0.5; 95% CI, 0.2 to 0.7) and FOXA2 (treatment naive: ρ = 0.5; 95% CI, 0.2 to 0.7; nCRT: ρ = 0.5; 95% CI, 0.3 to 0.8) was consistent across both treatment groups (Figure 2A). Correspondingly, GATA6 was co-expressed with the combination of E-cadherin/FOXA2 in all tumors (i.e., GATA6^high^E-cadherin^high^FOXA2^high^ or GATA6^low^E-cadherin^low^FOXA2^low^) except for a single instance in the treatment-naive group (Table S5). However, a diverging pattern between the treatment groups emerged when examining the correlation between GATA6 histoscores and the basal-like markers KRT5 and KRT17. In the treatment-naive group, GATA6 histoscores showed a significant negative correlation with KRT5 (ρ = −0.5; 95% CI, −0.7 to −0.2) and KRT17 (ρ = −0.5; 95% CI, −0.7 to −0.2) (Figure 2A). Consistently, GATA6 was mutually exclusive with basal-like markers in all treatment-naive tumors (i.e., GATA6^high^KRT5^low^KRT17^low^ or GATA6^low^KRT5^high^KRT17^high^) except for one instance (Table S5). However, within the nCRT group, there was no evident correlation between GATA6 histoscores and KRT5 or KRT17 (Figure 2A), and 50% of GATA6^high^ tumors co-expressed a combination of KRT5 and KRT17 (Table S5). Collectively, these findings provide robust evidence for GATA6’s potential as a biomarker to discriminate between classical and basal-like phenotypes in the treatment-naive setting. However, the conventional definitions of the classical and basal-like phenotypes may not persist following gemcitabine-based nCRT due to a subpopulation of tumors co-expressing GATA6 and KRT5/KRT17 emerging after treatment.

### GATA6-defined PDAC phenotypes prognosticate in treatment-naive tumors, but this association is lost in tumors pretreated with gemcitabine-based chemoradiotherapy

**Figure 3:**
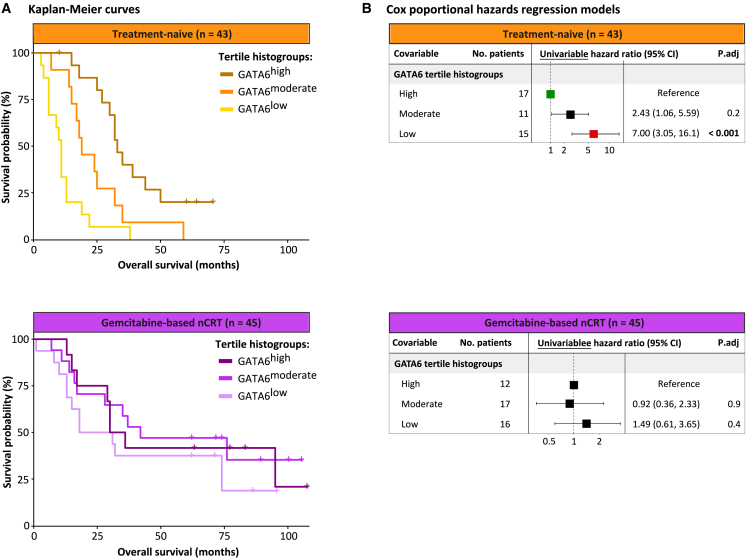
Survival analysis in patients with resected PDAC who underwent upfront surgery or gemcitabine-based nCRT (A) Kaplan-Meier curves, stratified by treatment and categorized by GATA6 histogroups, illustrate distinct OS outcomes associated with GATA6 in treatment-naive patients, contrasting with no associations between GATA6 and OS in nCRT-treated patients. Treatment-naive patients with GATA6^high^ tumors exhibit prolonged OS, while those with GATA6^low^ tumors exhibit reduced OS. The x axis displays the OS (months), and the y axis displays the survival probability (%). Cross symbols denote censored patients. (B) Forest plots, stratified by treatment, of the univariable and multivariable Cox proportional hazards regression models illustrate that a GATA6^low^ tumor phenotype is an unfavorable independent prognostic factor for OS in treatment-naive patients but not in nCRT-treated patients.

The prognostic utility of the GATA6 phenotypes in PDAC tumors was evaluated using unstratified and treatment-stratified Cox proportional hazards regression models. In treatment-naive patients, the univariable model revealed that those with GATA6^low^ tumors experienced significantly reduced OS (hazard ratio [HR] = 7.00; 95% CI, 3.05 to 16.1; *p* value adjusted for multiple testing [*p*.adj] < 0.001) (Figure 3). Interestingly, there was no evidence of an association between GATA6 phenotypes and prognosis in patients with PDAC treated with gemcitabine-based nCRT (Figure 3). In nCRT-treated patients, the univariable model showed no significant association between GATA6^low^ tumors and OS (HR = 1.49; 95% CI, 0.61 to 3.65; *p*.adj < 0.42). Multivariable models, stratified by treatment, were not computed since none of the other covariates emerged as significant prognostic factors for OS in univariable models (Table S6A). Moreover, adjusting for covariates measured after randomization (i.e., post-exposure variables) may introduce bias.^30^

The unstratified univariable model, including both treatment-naive and nCRT-treated patients, revealed that those with GATA6^high^ tumors experienced prolonged OS, albeit not statistically significant after adjustment for multiple testing (HR = 0.45; 95% CI, 0.25 to 0.80; *p*.adj = 0.091). After adjusting for treatment, the unstratified multivariable model revealed that patients with GATA6^high^ tumors experienced significantly prolonged OS (HR = 0.25; 95% CI, 0.13 to 0.48; *p*.adj < 0.001) (Table S6B).

The Cox proportional hazards regression models for PFS yielded consistent results with those observed for OS, both when stratified by treatment (Table S6C) and when unstratified (Table S6D).

### GATA6^high^ treatment-naive tumors overexpress epithelial identity-related genes and underexpress genes related to tumorigenesis

**Figure 4:**
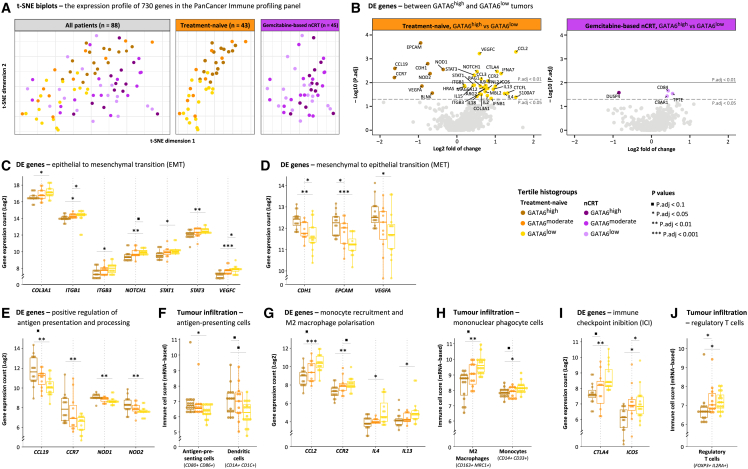
Transcriptomic NanoString nCounter analysis of treatment-naive and gemcitabine-based nCRT-treated PDAC tumors (A) t-SNE biplots illustrating the intra-tumoral expression profile of 730 genes in the PanCancer Immune profiling panel of all patients, treatment-naive patients, and nCRT-treated patients. Clear clusters of patients of distinct GATA6 histogroups are observed in the treatment-naive group, while no such clustering is present in patients who received nCRT. Each dot represents a patient, with coordinates depicting the first (x axis) and second (y axis) t-SNE dimensions. (B) Volcano plots, stratified by treatment, illustrate the differentially expressed (DE) genes between GATA6^high^ and GATA6^low^ tumors. The x axis displays the log2 fold of change, while the y axis displays the −log10 *p*.adj. Each dot represents a gene, and gene names indicate that they have exceeded the significance threshold of *p*.adj < 0.05. Genes on the right (positive) are overexpressed in GATA6^low^ tumors, and genes on the left (negative) are overexpressed in GATA6^high^ tumors. (C‒E, G, and I) Boxplots illustrating the log2 gene expression count (y axis) of DE genes between GATA6 histogroups (x axis). In GATA6^low^ compared to GATA6^high^ treatment-naive tumors, genes related to EMT (C), monocyte recruitment and M2 macrophage polarization (G), and ICI (I) are significantly overexpressed, whereas those related to MET (D) and positive regulation of antigen presentation and processing (E) are significantly underexpressed. (F, H, and J) Boxplots illustrating the mRNA-based immune cell score (y axis) of tumor-infiltrating immune cells (x axis). In GATA6^low^ compared to GATA6^high^ treatment-naive tumors, the tumor-infiltrating abundance of *CD80*^+^*CD86*^+^ antigen-presenting cells (significant) and *CD1A*^+^*CD1C*^+^ dendritic cells (not significant after adjustment for multiple testing) are impeded (F), while the tumor-infiltrating abundance of *CD163*^+^*MRC1*^+^ M2 macrophages, *CD14*^+^*CD33*^+^ monocytes, and *FOXP3*^+^*IL2RA*^+^ Tregs is significantly enhanced. In (C)–(J), each dot represents a patient.

The transcriptomic data, comprising 730 immuno-oncology-related genes of 88 surgical specimens, were explored using t-distributed stochastic neighbor embedding (t-SNE) dimensionality reduction analysis. Clusters of patients, including distinct GATA6 histogroups, were observed in the treatment-naive group but not in patients who received gemcitabine-based nCRT (Figure 4A). Accordingly, 36 genes were differentially expressed between GATA6^high^ and GATA6^low^ in treatment-naive tumors (Figure 4B). Among these genes, 28 were significantly underexpressed and eight were significantly overexpressed in GATA6^high^ tumors (Table S7). Genes related to tumorigenic pathways, including EMT, NOTCH, and JAK-STAT (e.g., *COL3A1*, *ITGB1*, *ITGB3*, *NOTCH1*, *STAT1*, *STAT3*, and *VEGFC*) were significantly underexpressed in GATA6^high^ treatment-naive tumors (Figure 4C). Conversely, genes associated with the epithelial phenotype and mesenchymal-to-epithelial transition (MET) (e.g., *CDH1*, *EPCAM*, and *VEGFA*) were significantly overexpressed in GATA6^high^ treatment-naive tumors (Figure 4D).

In contrast, only four genes were differentially expressed between GATA6^high^ and GATA6^low^ in nCRT-treated tumors (Figures 4B and S1). Immune profiling analysis for the GATA6^moderate^ phenotype can be found in Figure S2.

### GATA6^high^ treatment-naive tumors exhibit transcriptomic features associated with the activation of immunostimulatory mechanisms

We found no significant differences when comparing GATA6 histogroups among nCRT-treated tumors (Figure S3). In contrast, GATA6^high^ treatment-naive tumors showed significant gene overexpression in antigen presentation and processing (chemokine ligand 19 [*CCL19*], chemokine receptor 7 [*CCR7*], *NOD1*, *NOD2*) (Figure 4E). Correspondingly, *CD80*^+^*CD86*^+^ antigen-presenting cells were significantly more abundant in GATA6^high^ treatment-naive tumors (Figure 4F; *p*.adj = 0.04). A similar trend was observed for *CD1A*^+^*CD1C*^+^ dendritic cells, although the differences were not statistically significant after correction for multiple testing (Figure 4F, *p* = 0.02 and *p*.adj = 0.06).

GATA6^high^ treatment-naive tumors exhibited a significant underexpression of *CCL2* and *CCR2*, both genes coding proteins involved in orchestrating the recruitment of circulating monocytes (*p*.adj < 0.001 and *p*.adj = 0.009, respectively). Simultaneously, *IL4* and *IL13*, both of which play a crucial role in M2 macrophage polarization, were significantly underexpressed in GATA6^high^ treatment-naive tumors (Figure 4G; *p*.adj = 0.02 and *p*.adj = 0.03, respectively). As a corollary, *CD14*^+^*CD33*^+^ monocytes and pro-tumoral *CD163*^+^*MRC1*^+^ M2 macrophages were significantly less abundant in GATA6^high^ treatment-naive tumors (Figure 4H; *p*.adj = 0.04 and *p*.adj = 0.005, respectively).

Lastly, the immune checkpoint inhibitor (ICI) molecules cytotoxic T lymphocyte-associated protein (*CTLA4*) and inducible T cell co-stimulator (*ICOS*), often overexpressed in regulatory T cells (Tregs), were significantly underexpressed in GATA6^high^ treatment-naive tumors (Figure 4I; *p*.adj = 0.003 and *p*.adj = 0.01, respectively). Consistently, *FOXP3*^+^*IL2RA*^+^ Tregs were significantly less abundant in GATA6^high^ treatment-naive tumors (Figure 4J; *p*.adj = 0.03).

We conducted Spearman correlation analyses to examine the relationship between (continuous) GATA6 histoscores and immune cell counts, consistent with our findings based on GATA6 tertile histogroups (Figure S4). In nCRT-treated tumors, GATA6 histoscores exhibited no significant correlation with immune cell types. However, in treatment-naive tumors, GATA6 histoscores exhibited significant positive correlations with *CD80*^+^*CD86*^+^ antigen-presenting cells (ρ = 0.4; 95% CI, 0.1 to 0.6) and *CD1A*^+^*CD1C*^+^ dendritic cells (ρ = 0.4; 95% CI, 0.1 to 0.6) while demonstrating significant negative correlations with *CD14*^+^*CD33*^+^ monocytes (ρ = −0.4; 95% CI, −0.6 to −0.1), *CD163*^+^*MRC1*^+^ M2 macrophages (ρ = −0.3; 95% CI, −0.6 to −0.04), and *FOXP3*^+^*IL2RA*^+^ Tregs (ρ = −0.4; 95% CI, −0.6 to −0.2).

### Spatial protein profiling confirms the presence of immune cells associated with enhanced surveillance within treatment-naive GATA6^high^ tumors

To gain insight into the GATA6-associated immune landscape described above, surgical specimens from 24 treatment-naive patients with PDAC (11 GATA6^high^ and 13 GATA6^low^) underwent spatial protein profiling using the GeoMx digital spatial profiler (DSP), targeting 73 proteins relevant to immune-oncology processes. The clinical characteristics of these patients are provided in Table S8.

**Figure 5:**
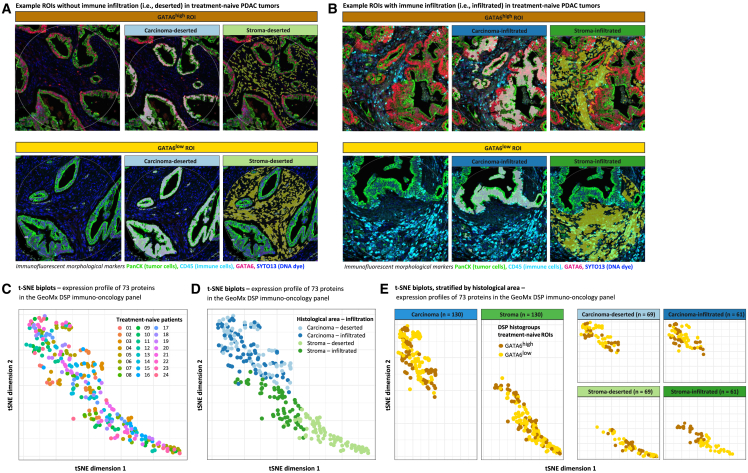
Digital spatial protein immune profiling NanoString GeoMx of treatment-naive PDAC tumors and explorative analysis (A and B) Microscopical images illustrating immunofluorescent staining with morphological markers for PanCK (epithelial tumor cells), CD45 (immune cells), GATA6, and SYTO13 (DNA dye) in treatment-naive tumors. CD45 expression classified ROIs into areas without immune infiltration (i.e., deserted and CD45 poor) (A) and areas with immune infiltration (i.e., infiltrated and CD45 rich) (B). Both images present three images for each DSP histogroup, illustrating the ROI without segmentation, with the carcinoma (PanCK-rich) and stroma (PanCK-poor) segments. (C‒E) t-SNE biplots illustrating the intra-tumoral expression profiles of 73 proteins in the GeoMx DSP immune-oncology panel in ROIs of treatment-naive patients. Each dot represents an ROI, with coordinates depicting the first (x axis) and second (y axis) t-SNE dimensions. No clustering of ROIs based on the individual patients is observed (C), while apparent clustering is observed based on the histological areas (D). After grouping by GATA6 (E), stroma-infiltrated ROIs showed an apparent clustering pattern, underscoring a possible association between GATA6 and immunological landscapes within this histological context.

The regions of interest (ROIs) were categorized into four histological areas using morphological markers (Figures 5A and 5B); 69 ROIs were classified as carcinoma deserted (Pan-cytokeratin [PanCK] rich/CD45 poor), 61 as carcinoma infiltrated (PanCK rich/CD45 rich), 69 as stroma deserted (PanCK poor/CD45 poor), and 61 as stroma infiltrated (PanCK poor/CD45 rich). The ROIs of the same histological classification were compared between GATA6^low^ and GATA6^high^ subgroups. The abundance of immune cell subtype markers was assessed relative to CD45 to account for interpatient variability in immune cell infiltration. However, CD163^+^ M2 macrophages were assessed relative to the total macrophage infiltration marker CD68 to allow the investigation of this specific subset within the broader macrophage population.

The selection of ROIs for immune profiling was unbiased, as evidenced by the t-SNE analysis, which revealed no discernible clustering of ROIs based on the individual patient (Figure 5C). Conversely, t-SNE analysis grouped by the four histological areas showed clear segregation of stroma-infiltrated and stroma-deserted areas (Figure 5D), suggesting the presence of distinctive immunological landscapes within these areas of stroma. The stroma-infiltrated ROIs showed an apparent clustering pattern upon GATA6-based grouping (Figure 5E), underscoring a possible association between GATA6 and distinct immunological landscapes within stroma-infiltrated areas.

**Figure 6:**
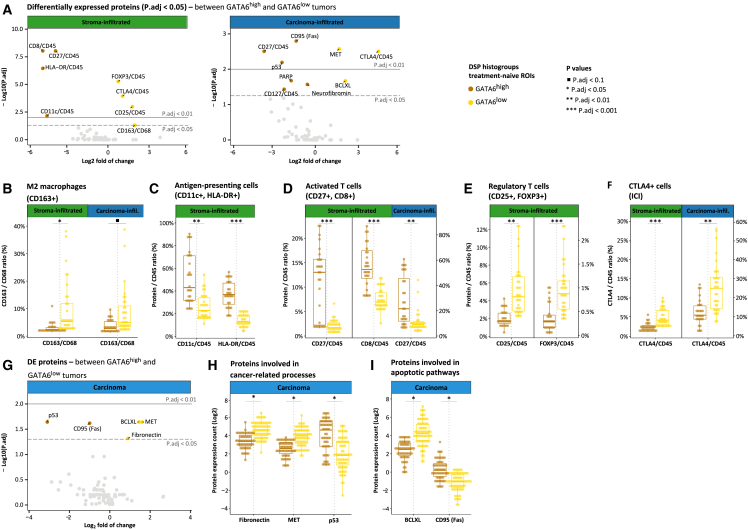
Digital spatial protein immune profiling NanoString GeoMx analysis of treatment-naive PDAC tumors (A and G) Volcano plots illustrate the DE proteins between GATA6^high^ and GATA6^low^ tumors. The x axis displays the log2 fold of change, while the y axis displays the -log10 *p*.adj. Each dot represents a protein, and protein names indicate that they have exceeded the significance threshold of *p*.adj < 0.05. Proteins on the right (positive) are overexpressed in GATA6^low^ tumors, and genes on the left (negative) are overexpressed in GATA6^high^ tumors. (B‒F) Boxplots illustrating the proportional abundance (y axis) of immune cell subtype markers relative to the general immune cell marker (CD45) in treatment-naive tumors. Notably, CD163^+^ M2 macrophages are evaluated relative to the total macrophage infiltration marker (CD68). Dual x axes account for disparities in data ranges, with distinct plots associated with the left and right x axes visually separated by the solid black line. The ratios of antigen-presenting cells (CD11c^+^, HLA-DR^+^) (C) and activated T cells (CD27^+^, CD8^+^) (D) are significantly lower, while the ratios of CD163^+^ M2 macrophages (B), regulatory T cells (CD25^+^, FOXP3^+^) (E), and CTLA4^+^ cells (F) are significantly higher in GATA6^low^ compared to GATA6^high^ stroma-infiltrated ROIs. The ratios of CD27^+^ activated T cells and CTLA4^+^ cells are significantly lower and higher, respectively, in GATA6^low^ compared to GATA6^high^ carcinoma-infiltrated ROIs. (H and I) Boxplots illustrating log2 protein expression counts (y axis) of proteins involved in cancer-related processes (H) and proteins involved in apoptotic pathways (I) in the carcinoma segment of treatment-naive tumors. The tumor-promoting fibronectin and MET and BCLXL anti-apoptotic proteins were significantly elevated, and the tumor-suppressing p53 and pro-apoptotic CD95 (Fas) proteins were significantly diminished in GATA6^low^ compared to GATA6^hgh^ carcinoma ROIs. In (E)–(K), each dot represents an ROI.

Consistent with the t-SNE analysis, the most prominent differences in immune infiltration were observed in stroma-infiltrated ROIs (Figure 6A). The ratio of M2 to total macrophages (CD163^+^/CD68^+^) was significantly lower in treatment-naive GATA6^high^ stroma-infiltrated ROIs (Figure 6B; *p*.adj = 0.04). A similar trend was apparent in treatment-naive GATA6^high^ carcinoma-infiltrated ROIs, although the differences were not significant after correction for multiple testing (Figure 6B; *p* = 0.01 and *p*.adj = 0.10). Furthermore, relative to CD45, CD11c^+^HLA-DR^+^ (human leukocyte antigen-DR) antigen-presenting cells and activated CD27^+^CD8^+^ T cells were significantly more abundant, coupled with significantly less abundance of CD27^+^CD8^+^ Tregs and CTLA4^+^ cells in GATA6^high^ stroma-infiltrated ROIs (Figures 6C–6F; all *p*.adj <0.01). Similar trends were observed in GATA6^high^ carcinoma-infiltrated ROIs, including increased CD27^+^ activated T cells and decreased CTLA4^+^ cells (Figures 6D and 6F; *p*.adj = 0.003).

Lastly, given the absence of clustering between carcinoma-deserted and carcinoma-infiltrated ROIs in the t-SNE analysis (Figure 5C), we merged both carcinoma areas to examine differences in GATA6^low^ and GATA6^high^ associated with epithelial cells in carcinoma ROIs, irrespective of immune infiltration (Figure 6G). The expression of fibronectin, a central component of the stiff extracellular matrix within the PDAC TME, and the proto-oncogene MET were significantly reduced in GATA6^high^ carcinoma ROIs (Figure 6H; *p*.adj = 0.04 and *p*.adj = 0.02, respectively). In contrast, the expression of the tumor-suppressor p53 was significantly elevated in GATA6^high^ carcinoma ROIs (Figure 6H; *p*.adj = 0.02). Furthermore, the expression of the anti-apoptotic BCLXL was significantly reduced, while the expression of the apoptotic protein CD95 was significantly elevated in GATA6^high^ carcinoma ROIs (Figure 6I; *p*.adj = 0.02 and *p*.adj = 0.03, respectively). These observed alterations in cellular processes predominantly promote tumorigenesis.

The immune subpopulations that showed significant transcriptomic differences but lacked statistical significance in the spatial protein analyses (including CD80^+^ antigen-presenting cells, CD14^+^ monocytes, and ICOS^+^ cells) are available in Figure S5. Notably, CD14^+^ monocytes displayed a similar trend and were less abundant in GATA6^high^ tumors.

## Discussion

In this post hoc analysis of the PREOPANC RCT, we validate the prognostic value of GATA6 IHC in treatment-naive patients with PDAC. Patients with GATA6^high^ tumors experience prolonged OS. However, the prognostic value of GATA6 IHC diminishes following gemcitabine-based nCRT, suggesting a declining ability of GATA6 to effectively distinguish PDAC subtypes after nCRT. Importantly, our findings suggest that treatment-naive PDAC tumors with varying GATA6 phenotypes manifest distinct immunological landscapes. GATA6 expression is associated with features indicative of enhanced immune surveillance. Transcriptomic and spatial protein immune profiling revealed increased infiltration of immune-stimulating activated T cells and antigen-presenting cells, coupled with restrained infiltration of immunosuppressive Tregs and M2 macrophages in GATA6^high^ tumors. These immunological distinctions within the TME may elucidate the improved survival observed in patients with GATA6^high^ tumors.

Although retrospective studies have previously highlighted the prognostic value of GATA6 IHC, linking lower GATA6 histoscores to unfavorable survival outcomes in both resected^14^^,^^17^ and advanced PDAC,^18^ it is crucial for prospective studies to substantiate these observations. This study, conducted within the confines of an RCT and a rigorously defined clinical framework, adds an extra layer of validation, emphasizing the imperative for continued exploration of GATA6 as a clinically relevant marker. Our findings reveal a positive correlation between GATA6 histoscores and classical phenotype markers (E-cadherin/FOXA2) while demonstrating a negative correlation with basal-like phenotype markers (KRT5/KRT17) in treatment-naive PDAC tumors. As anticipated, our survival analysis shows that treatment-naive patients with GATA6^high^ tumors experience prolonged OS. Conversely, GATA6 did not correlate negatively with basal-like cell markers in gemcitabine-based nCRT-treated tumors, aligning with previous findings.^13^^,^^19^^,^^25^ Correspondingly, the prognostic utility of GATA6-based PDAC phenotypes in these tumors was limited. Although molecular PDAC subtyping is promising to refine treatment selection and is being evaluated in two phase 2 clinical trials (ClinicalTrials.gov: NCT04469556 and NCT04683315), we caution that conventional PDAC phenotypes may not apply post-nCRT. This cautionary note aligns with the observations of Hwang et al., highlighting the remodeling of the pancreatic TME in response to treatment, including nCRT.^31^ Furthermore, different neoadjuvant approaches may have varying effects on the prognostic utility of GATA6-based PDAC phenotypes, with Kokumai et al. indicating that molecular PDAC subtypes could differentiate patient survival following neoadjuvant chemotherapy rather than chemoradiotherapy.^32^

GATA6 is a key transcription factor for acinar cell development,^33^ and it counteracts KRasG12V-induced tumorigenesis in murine models.^34^ In PDAC, it predominantly marks the classical subtype,^35^ while basal/squamous tumors may suppress GATA6 through transcriptional regulation, gene deletion,^13^ or promoter methylation.^36^ The loss of GATA6, although essential, is not the sole determinant for the transition to a basal-like program in PDAC.^26^ The absence of GATA6 triggers EMT, whereas its overexpression induces MET.^13^ Our study reaffirms the central role of GATA6 in determining PDAC phenotypes by illustrating that GATA6^low^ tumors exhibit transcriptomic alterations associated with a spectrum of tumorigenic processes. In GATA6^low^ tumors, EMT-related genes are overexpressed, linked to heightened tumor cell aggressiveness and invasiveness. In contrast, genes involved in the reverse process, MET, associated with restoring epithelial cell characteristics, are underexpressed in GATA6^low^ tumors. Spatial protein profiling revealed that GATA6^low^ tumors exhibit reduced expression of the tumor-suppressor p53 and elevated expression of the proto-oncogene MET and fibronectin, a crucial component of the extracellular matrix. These alterations could fuel PDAC tumorigenesis by encouraging processes such as EMT, immune evasion, and therapy resistance.^37^^,^^38^^,^^39^^,^^40^^,^^41^^,^^42^ However, the unavailability of p53 and KRAS mutation status in our study is noteworthy, as mutant p53 and KRAS are linked to increased aggressiveness and an immunosuppressive TME in PDAC.^40^ Furthermore, our spatial protein analysis reveals heightened anti-apoptotic BCL-XL expression and reduced apoptotic CD95 (Fas) expression in GATA6^low^ carcinoma areas. BCL-XL, commonly overexpressed in PDAC, plays a crucial role in disease progression by resistance to apoptosis and therapy.^43^^,^^44^ CD95, on the other hand, has a multifaceted role in cancer biology, affecting apoptosis, proliferation, and immune dynamics.^45^ These findings collectively suggest that GATA6-driven cellular phenotypes are not univocal.

Alongside its role in epithelial cell behavior, GATA6 may enhance anti-tumor immunity,^26^ supported by our immune profiling analysis. Firstly, a significantly higher abundance of *CD80*^+^ *CD86*^+^ antigen-presenting cells in GATA6^high^ tumors is observed, in parallel with the overexpression of genes involved in antigen presentation and processing.^46^^,^^47^ This observation aligns with previous studies.^26^ Our spatial protein profiling further confirms a significant enrichment in CD11c^+^ and HLA-DR^+^ antigen-presenting cells in GATA6^high^-infiltrated stroma areas. Secondly, GATA6^high^ tumors exhibit significantly decreased infiltration of *CD14*^+^ *CD33*^+^ monocytes and pro-tumoral *CD163*^+^ *MRC1*^+^ M2 macrophages. This may result from the underexpression of *CCL2* and *CCR2*, responsible for monocyte recruitment to tumor sites,^48^ and the reduced levels of *IL4* and *IL13*, genes known to promote monocyte polarization into M2 macrophages.^49^ Spatial protein profiling confirms a diminished abundance of infiltrating CD163^+^ M2 macrophages in GATA6^high^-infiltrated stroma areas. Tu et al. previously reported on the potential of patient-tailored immunotherapy affecting CCL2. They demonstrated that pharmacological inhibition of BRD4 suppressed the BRD4-cJUN-CCL2-tumor necrosis factor-α axis, restoring the classical subtype identity associated with a favorable prognosis.^50^ Thirdly, there is a significant decrease in immunosuppressive *FOXP3*^+^ *IL2RA*^+^ Tregs that infiltrated GATA6^high^ tumors, along with the underexpression of *CTLA4* and *ICOS,* both ICIs frequently overexpressed on Tregs.^51^ Spatial protein profiling corroborates these findings, revealing significantly decreased CD25^+^ and FOXP3^+^ Tregs in infiltrated stroma areas and CTLA4^+^ cells in both infiltrated stroma and carcinoma areas of GATA6^high^ tumors. Lastly, in concordance with previous literature,^26^ we observe a significant increase of CD27^+^ and CD8^+^ activated T cells in GATA6^high^-infiltrated stroma areas, displaying a similar significant trend for CD27^+^ T cells in infiltrated carcinoma areas. Grünwald et al. deciphered the pancreatic TME using integrated histology-guided multiOMICs, clinical data, and patient-derived preclinical models. In “reactive sub-TMEs,” tumor cells exhibited a more basal-like phenotype, evidenced by differentially expressed low GATA6. In addition to being more chemo-sensitive, these reactive sub-TMEs exhibited heightened immunostimulatory CD3^+^ and CD8^+^ T cell infiltration alongside elevated pro-tumoral IDO-1 and PD-L1 levels. Consistent with our observations, these sub-TMEs, associated with the basal-like subtype cancer cells, demonstrated increased expression of markers for FOXP3^+^ Tregs and CD206^+^ M2 macrophages.^28^ Taken together, our findings strongly suggest the presence of distinct immunophenotypes in treatment-naive primary PDAC tumors, each defined by varying levels of GATA6 expression. Specifically, GATA6^high^ tumors exhibit an immune-rich phenotype, while GATA6^low^ tumors manifest an immune-escaping phenotype.

In summary, the prognostic value of GATA6 IHC in resected tumors diminishes following gemcitabine-based nCRT. Furthermore, despite the absence of GATA6 knockout models, we found evidence that GATA6 may enhance immune surveillance in treatment-naive tumors. The TME in the absence of GATA6, characterized by reduced antigen presenting and activated T cell infiltration along with increased M2 macrophage and Treg infiltration, may well contribute to the poor survival observed in these patients. These insights provide a foundation for developing tailored and potent immune-based combination therapies in treatment-naive classical PDAC phenotypes.

### Limitations of the study

While our study leverages samples from an RCT, enhancing the credibility of our findings, there are limitations to consider. Firstly, our sample size, although valuable, may constrain the robustness of our statistical analyses. However, our clinical observations align with prior literature indicating prolonged survival in patients with GATA6^high^ tumors.^14^^,^^17^^,^^18^ Moreover, the prospective design of our study bolsters the reliability of our findings by ensuring temporal clarity, consistency in data collection, control over confounding variables, and long-term follow-up. Nevertheless, in this ancillary study, patients were not randomly selected from the PREOPANC trial but were chosen based on post-baseline events. This warrants caution, especially when interpreting the efficacy of neoadjuvant therapy in GATA6 subgroups. Secondly, the potential for interobserver variability in manual IHC scoring could impact result accuracy. This is a common concern in studies relying on manual scoring methods. Thirdly, the complex molecular landscape of PDAC extends beyond GATA6 expression, with the cooperation of GATA4 previously highlighted.^14^ We recognize multifaceted interactions among molecular drivers, TME components, and immune pathways that can influence the observed relationships. Employing pharmacological inhinitors or genetic modifications to knockout of GATA6 in PDAC models would have been preferred to more robustly validate our associations. Fourthly, emphasizing the notable intra-tumoral heterogeneity of GATA6 and classical/basal-like marker expression,^52^ our IHC analysis using one slide per tumor may only partially represent GATA6 diversity. This highlights the potential limitations in capturing the full spectrum of GATA6 expression within tumors. However, we addressed this limitation in our immune profiling analysis by employing spatial protein validation. We selected multiple ROIs per histological area and validated GATA6 IHC by evaluating a GATA6 immunofluorescent marker on separate slides for each tumor. Fifthly, translating our immune-based therapeutic insights targeting GATA6-associated immune alterations into clinical practice necessitates further exploration. Understanding the contributions of gemcitabine, radiotherapy, and their combination in modulating GATA6 functionality is essential. Matched pre-nCRT biopsies and post-nCRT samples could have provided valuable insights into how nCRT influences GATA6. Finally, as FOLFIRINOX remains the preferred preoperative treatment in many centers, notwithstanding recent findings from the PREOPANC-2 RCT suggesting comparable efficacy to gemcitabine-based nCRT,^12^ we plan to investigate various GATA6 phenotypes and their associated immunological profiles of neoadjuvant FOLFIRINOX-treated tumors.

## Consortia

The members of the Dutch Pancreatic Cancer Group are Daan J. Lips, Erwin van der Harst, Geert Kazemier, Gijs A. Patijn, Ignace H. de Hingh, Jan H. Wijsman, Joris I. Erdmann, Sebastiaan Festen, Bas Groot Koerkamp, J. Sven D. Mieog, Marcel den Dulk, Martijn W.J. Stommel, Olivier R. Busch, Roeland F. de Wilde, Vincent E. de Meijer, Wouter te Riele, I. Quintus Molenaar, Werner Draaisma, Eric Manusama, Kishan R.D. Lutchman, Susan van Dieren, Anniek Vlijm, Bert A. Bonsing, C. Yung Nio, Derik-Jan de Groot, Elske C. Gootjes, Eran van Veldhuisen, Fenny Wit, Freek Daams, Geert Cirkel, Geertjan van Tienhoven, Irene E.G. van Hellemond, Johanna W. Wilmink, Judith de Vos-Geelen, Koop Bosscha, Leonie J. Mekenkamp, Maarten W. Nijkamp, Maartje Los, Marion B. van der Kolk, Marjolein Homs, Mark Ramaekers, Mike S. Liem, Miriam L. Wumkes, Nynke Michiels, Ronald van Dam, Rutger T. Theijse, Saskia Luelmo, Thomas L. Bollen, Ulf Neumann, and Vincent Nieuwenhuijs.

## STAR★Methods

### Key resources table

**Table:** 

REAGENT or RESOURCE	SOURCE	IDENTIFIER
**Antibodies**		

E-cadherin	Ventana	Cat# 79044-97
Eosin	Abcam pic	Cat# ab2682
FOXA2	Seven Hills Bioreagents	Cat# wrap-1200
GATA6	R&D systems	Cat# AF1700
Hematoxylin	Abcam	Cat# ab220365
KRT5	Cell Marque	Cat# 760-4935
KRT17	Ventana	Cat# 790-4560
Pan-leukocyte (CD45), clone 2B11+PD7/26	Novus Biologicals	Cat# NBP2-34528
GATA6, clone Met1-Thr449	R&D systems	Cat# AF1700
Pan-cytokeratin (PanCK), clone AE1+AE3	Novus Biologicals	Cat# NBP2-33200
SYTO13	NanoString Technologies	Cat# 121303303

**Biological samples**		

Resected human PDAC tissues with or without nCRT	This study	PREOPANC; EudraCT 2012-003181-40

**Critical commercial assays**		

Ultraview DAB detection kit	Ventana	Cat# 760-500
Optiview DAB detection kit	Ventana	Cat# 760-700
RNeasy Plus Micro Kit	Qiagen	Cat# 74034
nCounter Standard Master Kit	NanoString Technologies	Cat# NAA-AKIT-048
nCounter PanCancer immune profiling panel	NanoString Technologies	Cat# XT-CSO-HIP1-12
GeoMx Solid Tumor TME Morphology Kit	NanoString Technologies	Cat# GMX-PRO-MORPH-HST-12
GeoMx Protein Slide Prep Kit for FFPE	NanoString Technologies	Cat# GMX-PREP-PRO-FFPE-12
GeoMx Immune Cell Profiling Panel	NanoString Technologies	Cat# GMX-PROCO-NCT-HICP-12
GeoMx IO Drug Target Panel	NanoString Technologies	Cat# GMX-PROMOD-NCT-HIODT-12
GeoMx Immune Activation Status Panel	NanoString Technologies	Cat# GMX-PROMOD-NCT-HIAS-12
GeoMx Immune Cell Typing Panel	NanoString Technologies	Cat# GMX-PROCO-NCT-MICP-12
GeoMx Pan-Tumor Panel	NanoString Technologies	Cat# GMX-PROMOD-NCT-HPT-12
GeoMx Cell Death Panel	NanoString Technologies	Cat# GMX-PROMOD-NCT-HCD-12
GeoMx PI3K/AKT Signaling Panel	NanoString Technologies	Cat# GMX-PROMOD-NCT-HPI3K-12
GeoMx MAPK Signaling Panel	NanoString Technologies	Cat# GMX-PROMOD-NCT-HMAPK-12

**Software and algorithms**		

2100 BioAnalyzer	Agilent Technologies	Model G2939B
Staining system Benchmark ULTRA	Ventana	Cat# N750-BMKU-FS 05342716001
QuPath	https://qupath.github.io/	v0.5.0
nCoutner Advanced Analysis 2.0	NanoString Technologies	V2.0 Cat# MAN-10030-03
GeoMx DSP Analysis Suite	NanoString Technologies	v2.1
R Statistical Software	https://cran.r-project.org	v4.1.2
ComplexHeatmap R package	https://rdocumentation.org/packages/ComplexHeatmap/versions/1.10.2	v1.10.2
corrplot R package	https://rdocumentation.org/packages/corrplot/versions/0.92	v0.92
EnhancedVolcano R package	https://rdocumentation.org/packages/EnhancedVolcano/versions/1.11.3	v1.11.3
ggplot2 R package	https://rdocumentation.org/packages/ggplot2/versions/3.4.4	v3.4.2
lme4 R package	https://rdocumentation.org/packages/lme4/versions/1.1-35.1	v1.1-32
Rtsne R package	https://rdocumentation.org/packages/Rtsne/versions/0.17	v0.17

### Resource availability

#### Lead contact

Further information and requests for resources and reagents should be directed to and will be fulfilled by the lead contact, Dana Mustafa (d.mustafa@erasmusmc.nl).

#### Materials availability

This study did not generate new unique reagents.

#### Data and code availability

• The raw datasets reported in this study are not deposited in a public repository to maintain patient consent standards and comply with ethical regulations. To request access under strict regulatory constraints, contact the Lead Contact Dana Mustafa (d.mustafa@erasmusmc.nl).
• This paper does not report original code. All codes used are publicly available and listed in the Key resources table.
• Any additional information required to reanalyze the data reported in this work paper is available from the Lead Contact Dana Mustafa (d.mustafa@erasmusmc.nl) upon request.

### Experimental model and subject details

#### PDAC patient cohort and clinical procedure

All patients in this study were recruited to the phase III randomized controlled PREOPANC trial (EudraCT number, 2012-003181-40).^10^ The current study only included patients with pathologically confirmed PDAC who completed the entire course of nCRT. The primary survival outcome in this study was OS, defined as the time from PDAC diagnosis to death. IHC analyses categorized the patient’s tumor tissues in GATA6^high^, GATA6^moderate^, and GATA6^low^ histogroups, and the same was done for classical phenotype markers (E-cadherin and FOXA2) and basal-like phenotype markers (KRT5 and KRT17). Inclusion and exclusion criteria can be found in the PREOPANC study protocol.^53^ After randomization, patients were assigned to undergo upfront surgery within four weeks (i.e., treatment-naive) or receive gemcitabine-based nCRT followed by surgery within four to six weeks. Patients in the nCRT group underwent a staging laparoscopy before treatment, followed by three cycles of gemcitabine (1000 mg/m^2^) combined with hyperfractionated radiotherapy (36 Gy) in 15 fractions during the second cycle. Patients from both study arms underwent resection only if there were no metastases or locally unresectable diseases at the time of surgery. Adjuvant gemcitabine (1000 mg/m^2^) was scheduled to be administered within 12 weeks after surgery, with four cycles for the nCRT group and six for the upfront surgery group. Surgical specimens were formalin-fixed paraffin-embedded (FFPE) and retrieved following the International Study Group consensus statement on Pancreatic Surgery.^54^ The primary outcome in this study was OS, defined as the time from PDAC diagnosis to death.

#### Ethics approval and consent to participate

The participating patients in this study originated from the phase III PREOPANC RCT (EudraCT 2012-003181-40) performed in 16 high-volume pancreatic surgery centers from the Dutch Pancreatic Cancer Group (DPCG). This trial was conducted according to the guidelines of the Declaration of Helsinki and approved by the Ethics Committees of Erasmus MC (MEC-2012-249; December 11, 2012). Written informed consent was obtained from all patients.

### Method details

A schematic overview of the methodological steps can be found in Figure 1.

#### Immunohistochemistry staining

Four-micron sections of FFPE samples were stained with hematoxylin and eosin using HE600 according to the manufacturer’s instructions (Ventana Medical Systems Inc., Arizona, USA). Immunohistochemistry was performed with an automated, validated, and accredited staining system (Ventana Benchmark ULTRA, Ventana Medical Systems) using the ultraview (#760-500, Ventana) or optiview (#760-700, Ventana) universal DAB detection kit. In brief, following deparaffinization and heat-induced antigen retrieval, tumor cell-rich sections of 5μm from the FFPE blocks were immunostained for GATA6, classical phenotype markers (E-cadherin and FOXA2), and basal-like phenotype markers (KRT5 and KRT17), followed by hematoxylin counterstain. Incubation was followed by a hematoxylin II counter stain for 20 min and a blue coloring reagent for 8 min according to the manufacturer’s instructions (Ventana Medical Systems Inc., Arizona, USA). Histoscores were determined using a modified semi-quantified histochemical scoring method, as previously described.^55^^,^^56^ In short, a specialized pancreatic pathologist (MD) identified tumor-cell-rich regions in hematoxylin and eosin-stained slides. Subsequently, four independent observers (CWFvE, DAMM, FXR, and MD) evaluated the expressions of the different antibodies within these tumor-cell-rich regions. The final histoscore was calculated by multiplying the proportion of stained tumor cells by an ordinal value (ranging from 0 to 3), representing the intensity of the staining. Importantly, tertile groups were calculated based on the histoscores in the entire cohort to avoid obscuring any differences in GATA6 histoscore distribution between treatment-naive and nCRT-treated tumors. Some samples stained for GATA6 lacked concurrent staining for other classical and basal-like markers due to sample availability.

#### Targeted transcriptomic immune profiling using NanoString technologies

To gain insights into the immunological landscape associated with GATA6 expression in PDAC tumors, targeted gene expression profiles of the surgical specimens were reanalyzed and generated as described previously.^29^ Tissue sections with a thickness of 5 μm were cut from the FFPE PDAC tumor blocks. Following deparaffinization and staining with hematoxylin and eosin, an expert pancreatic pathologist (MD) identified tumor-cell-rich regions within the sections and examined the slides for tumor cellularity. RNA isolation from these selected regions was performed using the RNeasy Plus Micro Kit (Qiagen, Hilden, Germany), following the manufacturer’s instructions. The number of sections used for RNA isolation was adjusted considering tumor size and cellularity variations. The quality of the isolated RNA was assessed using the Agilent 2100 BioAnalyzer (Santa Clara, CA, USA), and concentration adjustments were made to account for RNA degradation. The nCounter PanCancer immune profiling panel, developed by NanoString Technologies (Seattle, WA, USA), was used for targeted multiplex gene expression profiling of the tissue RNA. This panel encompasses 730 immuno-oncology-relevant, 40 housekeeping, six positive control, and eight negative control genes (Table S9). Its design quantifies the relative abundance of immune cell subtypes, immune checkpoints, and chemokines associated with innate and adaptive immune responses.

Each sample, containing 300 ng RNA in a maximum volume of 7 μL, underwent hybridization at 65°C for 17 h using the SimpliAmp Thermal Cycler (Applied Biosystems). Subsequently, the nCounter FLEX instrument quantified the gene expression levels by scanning 490 fields of view.^57^ The raw gene expression data underwent quality checks, followed by normalization and log2 transformation using the Advanced Analysis module (v2.0) of NanoString nSolver software (v4.0).^58^ Normalization was conducted using the geNorm algorithm based on the stable housekeeping genes.^59^ Downstream analysis was conducted exclusively on genes that surpassed the detection limit in more than 80% of the samples. The detection limit was twice the average expression level of all negative controls.

The relative abundance of intra-tumoral immune cell subtypes between tumors of different GATA6 histogroups was compared using marker genes from the PanCancer Immune profiling panel tailored to represent cell types in PDAC accurately.^60^ Immune cell-specific candidate markers with a pairwise similarity (R^2^) of ≥0.6 were considered representative of a specific immune cell type (Table S10). The cell type score is the average of the marker gene expression values.

#### Digital spatial protein immune profiling

Sections with a thickness of 5 μm were cut from the FFPE tumor blocks of treatment-naive PDAC patients. Following deparaffinization, sections were simultaneously incubated with four immunofluorescent morphological antibodies and the immuno-oncology protein panel consisting of 78 immuno-oncology-related, three housekeeping, and three negative control target proteins (Table S11). Subsequently, stained sections were loaded onto the GeoMx digital spatial profiler (DSP) of NanoString Technologies and scanned according to the manufacturer’s instructions,^61^ as previously described in detail.^29^

Sample selection for DSP was guided by GATA6 IHC expression, aiming for tumors with distinct high or low GATA6 levels. Regions of interest (ROIs) selection was guided by four fluorescent DSP morphological markers, including GATA6, Pan-cytokeratin (PanCK) for epithelial tumor cells, and pan-leukocyte marker (CD45) for all hematopoietic cells, accompanied by the DNA dye SYTO13 to confirm the presence of nuclei within the selected ROIs. GATA6 expression was used to classify these ROIs into GATA6^high^ and GATA6^low^. Pan-cytokeratin (PanCK) expression was used for segmentation into areas of carcinoma (PanCK-rich) and stroma (PanCK-poor). CD45 expression further classified the ROIs into immune-infiltrated (CD45-rich) or immune-deserted (CD45-poor) ROIs. After a digital image of the fluorescent morphological markers was produced, three replica ROIs per histological area for each patient were selected to account for intra-tumoral heterogeneity. Not all tumors contained both areas with and without immune infiltration, resulting in deficient ROI numbers. Various geometric shapes, including polygons and circles, were used for ROI selection.

After quantifying antibody expression using the nCounter platform, the raw protein expression data were accessible within the GeoMx DSP Analysis Suite (v2.1). Data quality control and normalization were performed in the Analysis Suite to enable comparison across ROIs with varying sizes and cell numbers. Normalization was performed using the positive controls from the External RNA Control Consortium, followed by normalization based on the two stable housekeeping proteins, ribosomal protein S6, and histone H3. In addition, background expression correction was done based on the negative control Ms IgG2a. The normalized data were ultimately exported from the GeoMx DSP Analysis Suite for downstream analysis.

#### Data exploration using dimensionality reduction

We performed t-SNE dimensionality reduction analysis on the gene expression and spatial protein datasets to explore potential GATA6 histogroup clustering patterns driven by the TME immune composition. This approach preserves the structure of the data points while visualizing the high-dimensional datasets in a two-dimensional space. A simple in-between-group comparison (GATA6^high^ vs. GATA6^low^) may conceal such clustering, especially if the TME of a subset of GATA6^high^ tumors behaved like that of GATA6^low^ tumors or vice versa. The t-SNE was optimized with the hyperparameters (‘perplexity’ = 10, ‘max_iter’ = 5000, and ‘theta’ = 0).

### Quantification and statistical analysis

Downstream statistical analyses and data visualizations were performed in R Statistical Software (v4.1.2) using the ’ComplexHeatmap’ (v1.10.2), ‘corrplot’ (v0.92 ‘EnhancedVolcano’ (v1.11.3), ‘ggplot2’ (v3.4.2), ‘lme4’ (v1.1-32), ‘Rtsne’ (v0.16), and ‘survival’ (v3.5-5) packages. Preoperative clinicopathological characteristics and postoperative outcomes were analyzed using Fisher’s exact test for categorical variables and Mann-Whitney U test for continuous variables. Survival outcomes were estimated using the Kaplan-Meier method, and patients were censored if they remained alive at the last follow-up. The impact of clinicopathological characteristics and histoscores on survival outcomes was evaluated using Cox proportional hazards regression models. The proportional hazards assumption was assessed for each covariable, with no violations observed (*p* > 0.05).

The correlation between the staining patterns of different histochemical markers was assessed using Spearman correlations. Gene expression and immune cell subtypes were compared between groups using non-parametric unpaired Mann-Whitney U tests (i.e., Wilcoxon rank-sum test). DSP data was statistically tested between groups using linear mixed models to account for repeated ROIs per patient, following the manufacturer’s guidelines.^61^ *p* values <0.05 were considered statistically significant and adjusted for multiple testing by calculating the false discovery rate using the Benjamini-Hochberg correction (P.adj) if necessary. The Benjamini-Hochberg correction was applied individually for each analysis, ensuring specificity to their characteristics and assumptions.

## Supplemental information

Document S1. Figures S1‒S5

Table S1. Surgery type and clinicopathological outcomes of the 88 included patients with resected PDAC, related to Table 1

Table S2. The clinical characteristics of the 88 patients with resected PDAC stratified by GATA6, related to Table 1

Table S3. The clinical characteristics of the 75 patients with resected PDAC are included in the IHC analysis, related to Figure 2

Table S4. Results of immunohistochemistry analysis in the surgical specimens of 75 included patients with PDAC, related to Figure 2

Table S5. Results of the GATA6 and classical and basal-like phenotype IHC analysis, related to Figure 2

Table S6. Unstratified and stratified Cox regression models of the 88 patients with resected PDAC, related to Figure 3 A) Stratified univariable Cox proportional hazards regression models, OS since diagnosis. B) Unstratified Cox proportional hazards regression model, OS since diagnosis. C) Stratified univariable Cox proportional hazards regression models, PFS since diagnosis. D) Unstratified Cox proportional hazards regression models, PFS since diagnosis

Table S7. Transcriptomic NanoString analysis, differentially expressed genes (*p*.adj < 0.05) between GATA6^high^ and GATA6^low^ tumors in both treatment groups, related to Figure 4

Table S8. Clinical characteristics of the 24 patients with resected PDAC included in the DSP analysis, related to Figure 5

Table S9. NanoString nCounter PanCancer Immune profiling panel (770 genes), related to the STAR Methods

Table S10. Selected marker genes to identify immune cell types, related to the STAR Methods

Table S11. GeoMx immuno-oncology protein panel (78 proteins), related to the STAR Methods

Document S2. Article plus supplemental information
