# Supplementary material for: GATA6 identifies an immune-enriched phenotype linked to favorable outcomes in patients with pancreatic cancer undergoing upfront surgery
Source: Cell Rep Med. 2024 May 10;5(5):101557. doi: 10.1016/j.xcrm.2024.101557 (PMC11148804; doi:10.1016/j.xcrm.2024.101557)
Supplement: Document S1. Figures S1‒S5 [file mmc1.pdf]

Cell Reports Medicine, Volume *5*

Supplemental information

GATA6 identifies an immune-enriched phenotype linked to favorable outcomes in patients with pancreatic cancer undergoing upfront surgery

Casper W.F. van Eijck, Francisco X. Real, Núria Malats, Disha Vadgama, Thierry P.P. van den Bosch, Michail Doukas, Casper H.J. van Eijck, Dana A.M. Mustafa, and the Dutch Pancreatic Cancer Group (DPCG)

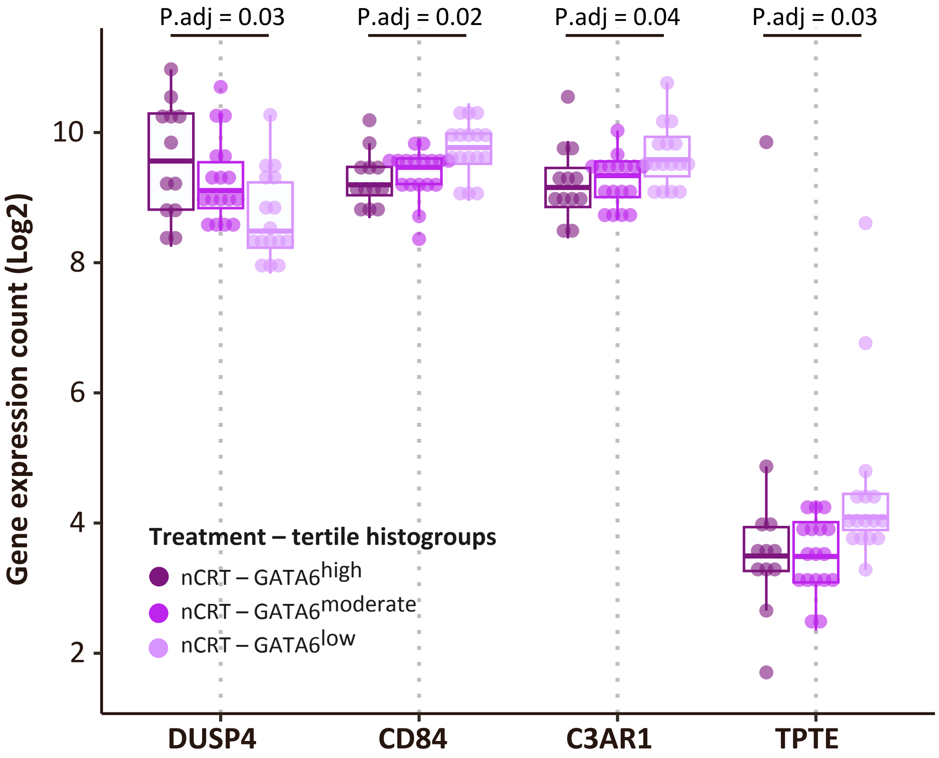

Figure S1. DE genes between gemcitabine-based nCRT-treated GATA6^{high} and GATA6^{low} tumors, related to Figure 4.

Boxplots illustrating the log2 gene expression count (y-axis) of DE genes between GATA6 histogroups (x-axis) of tumors treated with nCRT. Each dot represents a patient. nCRT, neoadjuvant Chemoradiotherapy; P.adj, P value adjusted for multiple testing using the Benjamini-Hochberg correction.

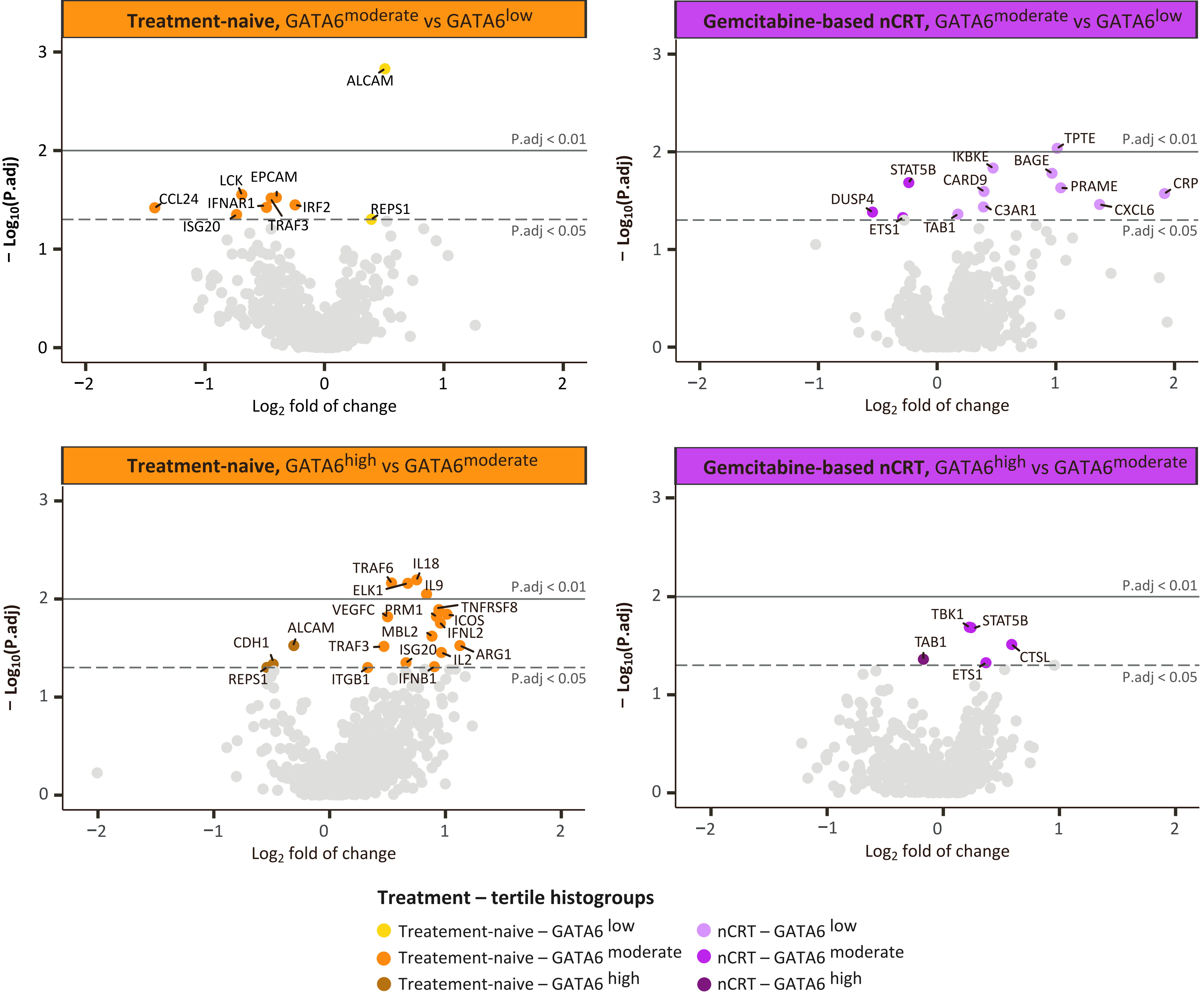

Figure S2. DE genes between GATA6moderate and GATA6low or GATA6high PDAC tumors, related to Figure 4.

Volcanoplots are stratified by treatment group (treatment-naive or gemcitabine-based nCRT). The x-axis displays the log2 fold of change, while the y-axis displays the -log10 P.adj. Each dot represents a gene, and gene names indicate that they have exceeded the significance threshold of P.adj < 0.05. DE, Differentially Expressed; Log, Logarithmic; nCRT, neoadjuvant Chemoradiotherapy; P.adj, P value adjusted for multiple testing using the Benjamini-Hochberg correction; PDAC, Pancreatic Ductal Adenocarcinoma.

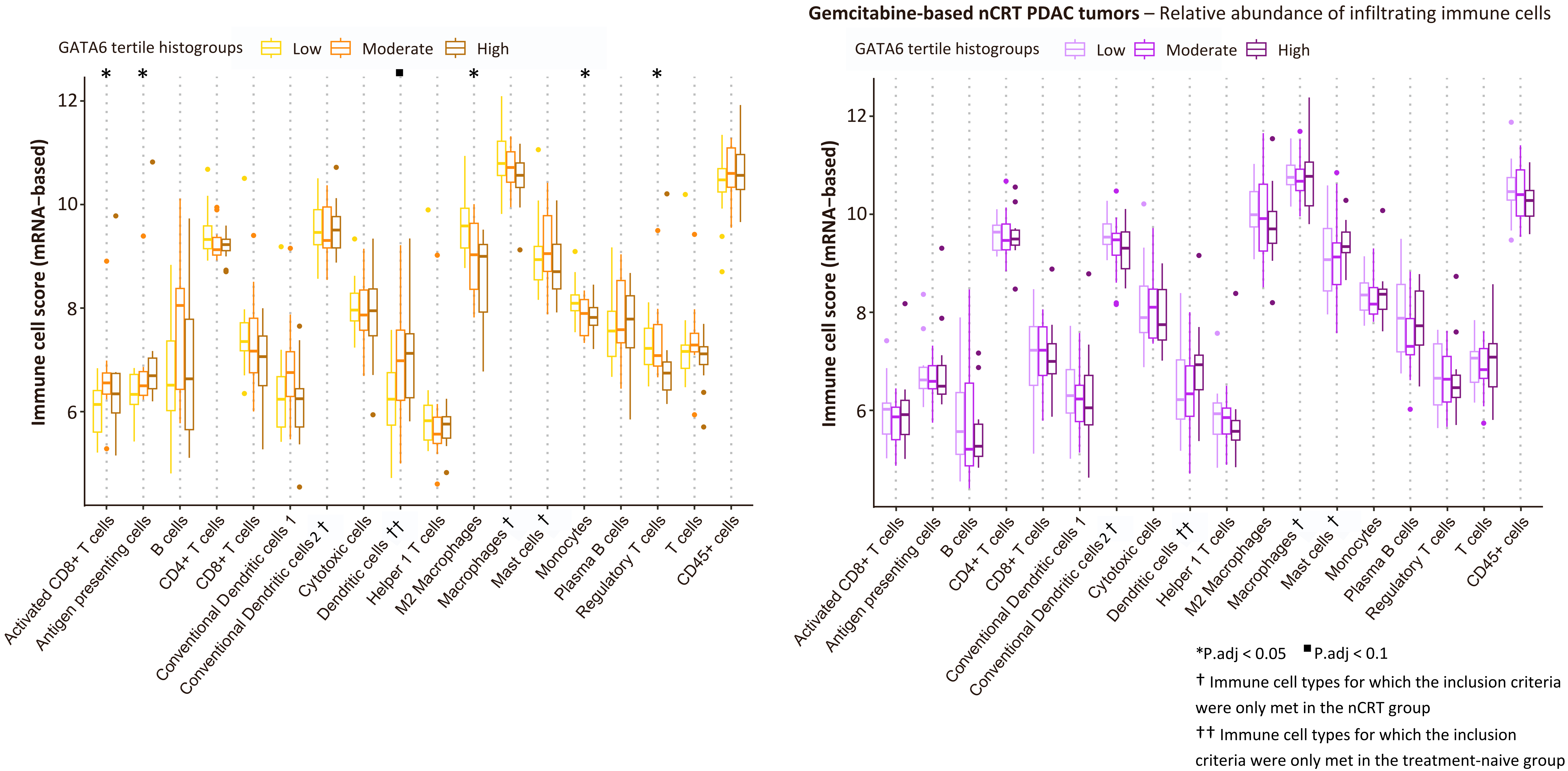

Figure S3. The abundance infiltrating immune cells, quantified by mRNA marker expression, in PDAC tumors of varying GATA6 histogroups, related to Figure 4.

Boxplots are stratified by treatment group (treatment-naive or gemcitabine-based nCRT) and classified based on GATA6 tertile histogroups. The x-axis displays the different immune cell types, and the y-axis displays the immune cell type score based on corresponding marker gene expression. Single dagger denotes immune cell types for which the inclusion criteria were only met in the nCRT group. Double daggers denote Immune cell types for which the inclusion criteria were only met in the treatment-naive group. CD, Cluster of differentiation; nCRT, neoadjuvant Chemoradiotherapy; P.adj, P value adjusted for multiple testing using the Benjamini-Hochberg correction; PDAC, Pancreatic Ductal Adenocarcinoma.

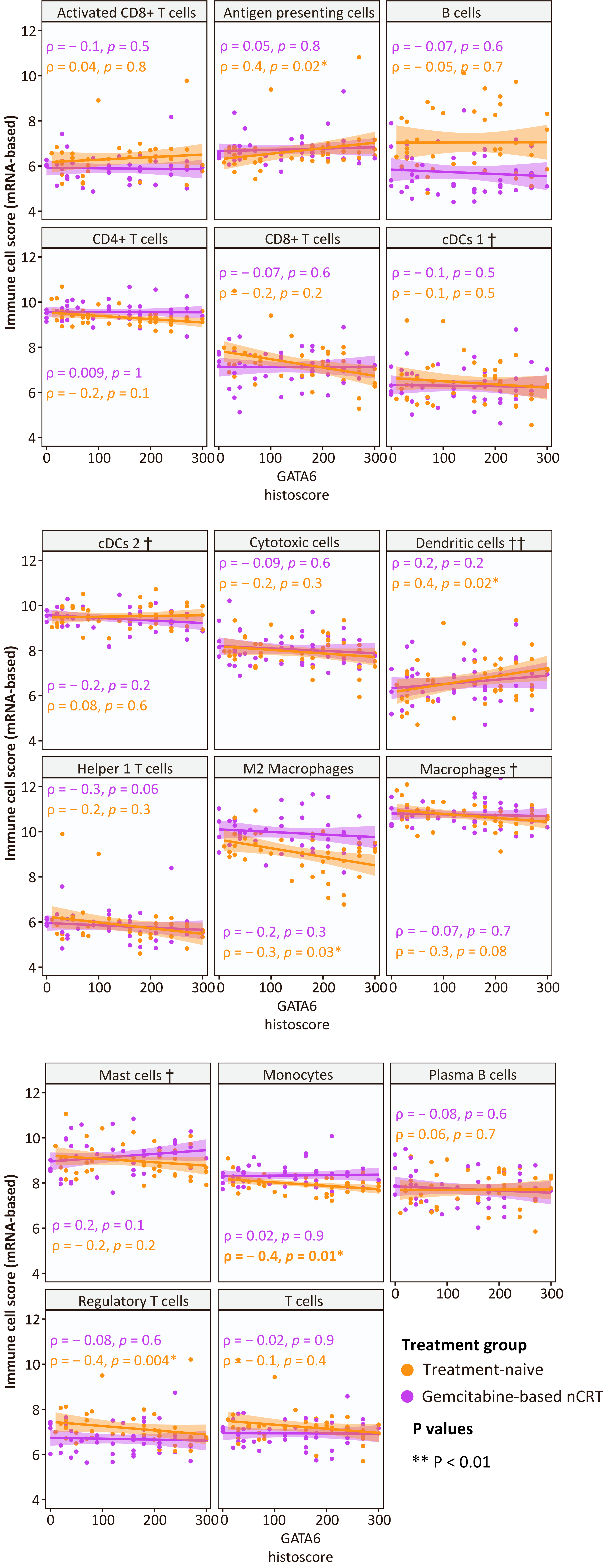

Figure S4. Spearman correlations between the immune cell scores and GATA6 histoscores of resected PDAC tumors, stratified by treatment, related to Figure 4.

Scatterplots illustrating Pearson's correlations between the GATA6 histoscores (x-axis) and immune cell scores (y-axis), stratified by treatment. Each dot represents a patient. Correlation coefficients are denoted with ρ with 95% confidence intervals, and '*' Indicates statistical significance at $P < 0.05$. † Indicates immune cell types for which the inclusion criteria were only met in the nCRT group. †† indicates immune cell types for which the inclusion criteria were only met in the treatment-naive group. nCRT, neoadjuvant Chemoradiotherapy; PDAC, Pancreatic Ductal Adenocarcinoma.

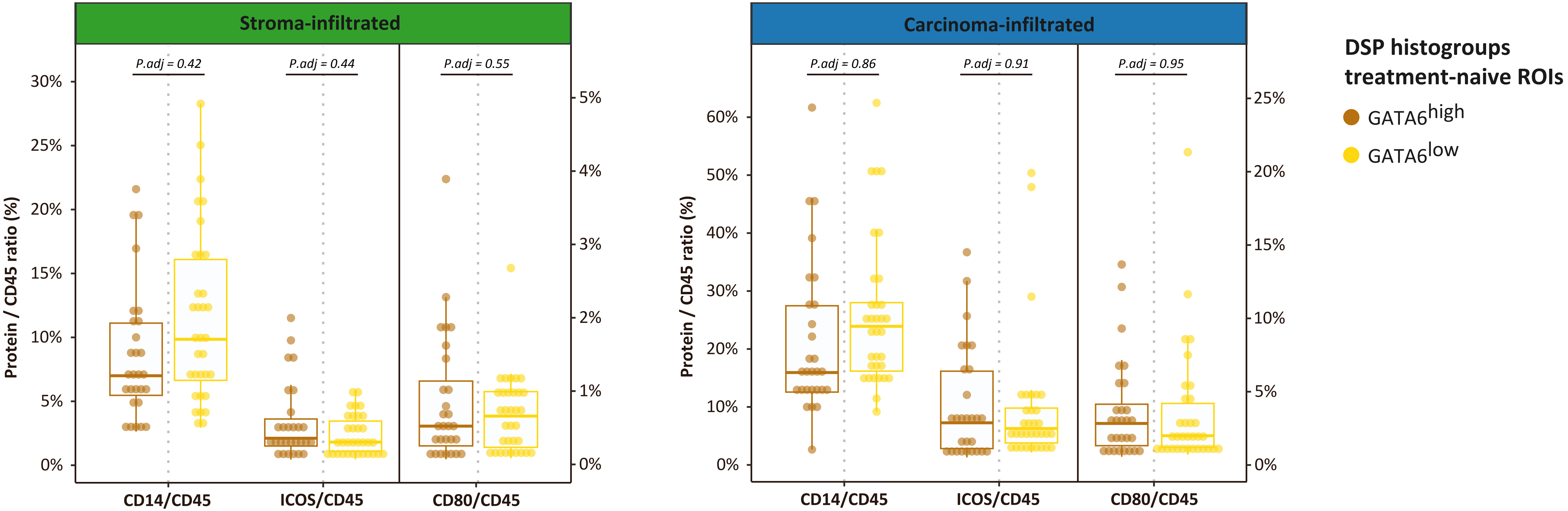

Figure S5 Boxplots of the proportional abundance of CD14+ monocytes, ICOS+ cells, and CD80+ antigen-presenting cells relative to the general immune cell marker (CD45) in treatment-naive PDAC tumors, related to Figure 6.

Boxplots are stratified by stroma-infiltrated (PanCK-poor CD45-rich) and carcinoma-infiltrated (PanCK-rich CD45-rich) histological areas and classified based on GATA6 DSP histogroups. The x-axis displays the different immune cell type marker proteins, and the y-axis displays the protein-to-CD45 ratio. Dual x-axes account for disparities in data ranges, with distinct plots associated with the left and right x-axes visually separated by the solid black line. Each dot represents an ROI. CD, Cluster of differentiation; P.adj, P value adjusted for multiple testing using the Benjamini-Hochberg correction; ICOS, Inducible T-cell Co-Stimulator; PDAC, Pancreatic Ductal Adenocarcinoma. ROI, Region of Interest.
